# Bloom Syndrome Complicated by Low-Grade Lymphoma and Non-small Cell Lung Cancer: A Case Report

**DOI:** 10.7759/cureus.60107

**Published:** 2024-05-11

**Authors:** Nathan J. Gould, Emma J.B. Holjak, Jalal Barat, Keng Yeow Tay, A. Rashid Dar

**Affiliations:** 1 Schulich School of Medicine and Dentistry, Western University, London, CAN; 2 Radiology, Victoria Hospital, London, CAN; 3 Radiology, London Health Sciences Centre, London, CAN; 4 Radiation Oncology, London Health Sciences Centre, London, CAN

**Keywords:** radiotherapy, paclitaxel, radiosensitivity, b-cell lymphoma, genetic disorder, nsclc, bloom syndrome

## Abstract

Bloom syndrome (BS) is a rare autosomal recessive genetic disorder characterized by photosensitivity, rashes on the nose and cheeks, short stature, and a predisposition to develop cancers. In this report, we discuss the diagnosis and management of a 34-year-old Canadian male BS patient, originally from Honduras, who developed B-cell lymphoma and a subsequent non-small cell lung carcinoma (NSCLC).

Given the radiosensitivity of the patient due to his BS diagnosis and the early stage of the low-grade B-cell lymphoma, we relied on surveillance as the clinical approach to his management. The treatment for NSCLC was initiated in stage III of the disease and was palliative in intent. Chemotherapy (12 rounds of paclitaxel, with the dosage gradually increasing from 48 mg to 58 mg and finally to 72 mg) was employed to shrink the left upper lobe (LUL) lung mass. Subsequently, radiotherapy (3000 cGY in 20 fractions) was administered to improve symptoms further. The radiotherapy dose schedule was modified given the patient’s BS diagnosis to avoid excessive toxicity. The palliative treatment course was well tolerated by the patient and resulted in symptom relief. However, his cancer progressed over the course of the treatment, ultimately resulting in his death 18 months after the initial diagnosis of NSCLC; no autopsy was performed. We believe this report will spur clinicians to engage in fruitful discussions about tailoring chemotherapy and radiation therapy regimens for treating cancer in BS patients.

## Introduction

Bloom syndrome (BS) is a rare genetic disorder, first described in 1954 by Dr. David Bloom [1]; it is characterized clinically by photosensitivity, rashes on the nose and cheeks, and short stature. Its complications include early-onset immune deficiencies and increased susceptibility to diseases such as cancer, chronic obstructive pulmonary disease (COPD), and diabetes. So far, less than 300 cases of BS have been reported in the BS registry [2]. It is a monogenic, autosomal recessive genetic disorder caused by mutations in both alleles of the BLM gene, which is an essential gene in DNA repair and growth. The most common BS-causing mutation is BLM^Ash ^[3], which involves a 6-bp deletion and 7-bp insertion at nucleotide position 2281 in the BLM gene [4].

At the genomic level, BS is characterized by chromosomal instability, including a high number of spontaneous chromosomal breakages, excessive homologous recombination, and increased rates of sister chromatid exchange. In healthy individuals, the BLM gene encodes RecQL3, which is an ATP-dependent DNA helicase known as a “genome caretaker” for the critical roles it plays in DNA replication, transcription, and recombination. Additionally, BLM is considered a tumor suppressor gene as it regulates the expression of both oncogenes and tumor suppressor genes, thus inhibiting tumorigenesis at early stages. As a result, individuals with BS are more likely to develop cancer, with 50% of BS patients developing cancer in their lifetimes [2]. 

While cancer diagnoses in patients with BS have a similar distribution to that of the general population, BS patients present with cancer at a much earlier age than what is typically seen in the general population. Additionally, individuals with BS are more likely to have more than one type of cancer (multiple primaries) and more likely to have rarer types of cancers [5]. While a wide range of cancers have been documented in individuals with BS, leukemia, lymphoma, and malignancies of the gastrointestinal tract are the most common types. Given that cancer is the most common cause of death in BS patients, prevention and screening are critical in this patient population. Extensive guidelines have been published outlining how patients with BS should be counseled regarding both preventative measures and the cancer screening required for patients throughout their lifespan [6]. In individuals with BS, the treatment of cancer is additionally complex as the physicians need to treat their cancer without unnecessarily exposing patients to agents that could trigger additional cancer development and progression in the future [7].

## Case presentation

The patient was a 34-year-old male with BS who developed B-cell lymphoma and subsequent non-small cell lung carcinoma (NSCLC) of the left upper lobe (LUL) of the lung. The diagnosis of BS was delayed as the patient and his family had primarily lived in refugee camps in Honduras where he was born, likely without access to adequate healthcare. In 1990, the patient and his family came to Canada as refugees. At the age of 13, he presented to the hospital with short stature. His mother reported that he had stopped growing at the age of 10 years and he was found on examination to have facial erythema, microcephaly, and delayed mental development. The patient was evaluated by medical genetics and a skin biopsy for cytogenetics was taken from his left arm; 103 cells were examined and showed the presence of three cell lines. Cultured fibroblasts by fluorescent banding showed a male [45, X/46, XY with multiple breaks and gaps/46, XY, t (5q-;14q+)] with mosaic chromosome complement. Overall, a significant number of chromosomal breaks and re-arrangements were present, and the patient was diagnosed with BS.

The patient was involved in a motor vehicle accident as a pedestrian and subsequently presented to a trauma follow-up clinic in January 2011, where they discovered a new mass on the left side of his neck. On physical examination, it was described as a mobile, 2-3 cm lymph node in the left posterior cervical chain that was tender to palpation. A fine-needle aspiration biopsy was done, and the findings were suggestive of a reactive process (i.e., numerous and variable lymphocytes, neutrophils, and histiocytes). In February 2011, a Tru-Cut core needle biopsy was taken from the lymph node for flow cytometric testing to confirm the absence of a B-cell lymphoproliferative disorder. However, the biopsy stained positive for CD20 and was suggestive of a B-cell lymphoproliferative disorder but there was not any definitive evidence of clonality based on kappa lambda staining.

Given his increased risk of lymphoma due to BS, staging investigations were performed (CT chest, abdomen, and pelvis) and bone marrow aspirate biopsy and cytogenetics were ordered to look for more conclusive evidence of a B-cell malignancy. The bone marrow aspirate, flow cytometry, and cytology were normal. The CT chest showed a 1.6 cm stable hilar lymph node as well as some non-pathological lymph nodes that did not meet the size criteria. Pulmonary nodules were noted but they were relatively unchanged and subcentimetric from his previous investigations. Overall, it was decided that the pathology from the left neck node biopsy was not completely conclusive but seemed to suggest low-grade B-cell lymphoma. At this time, the lymph node was too small to excise for definitive staging, and given his underlying BS diagnosis and associated radiosensitivity, it was decided that surveillance would be the best course of treatment. 

**Figure 1:**
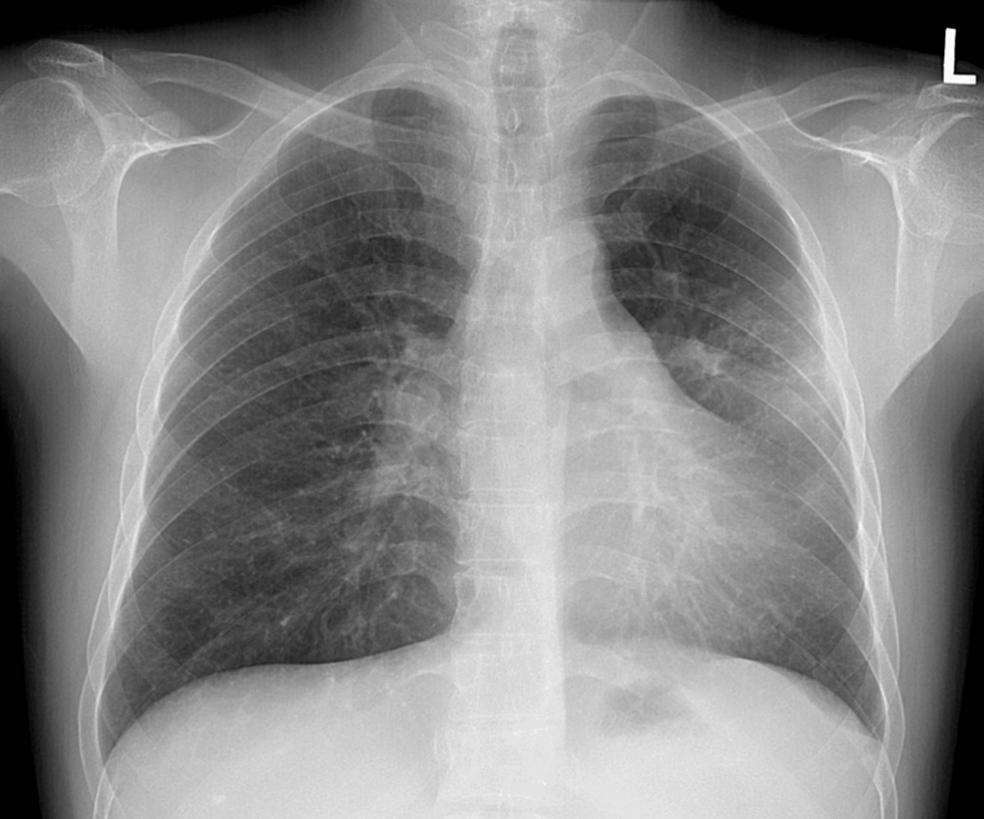
AP chest X-ray depicting LUL opacity (October 18, 2011) AP: anteroposterior; LUL: left upper lobe

Nine months later, in October 2011, the patient presented with a one-month history of a persistent cough and yellow phlegm with no hemoptysis. He had significant dyspnea, which limited him from walking one block. The patient had a 14-pack-year smoking history but he had quit in 2010. A chest X-ray revealed hilar enlargement, vague left midlung opacity, and a slightly smaller left lung suggestive of early volume loss (Figure 1).

**Figure 2:**
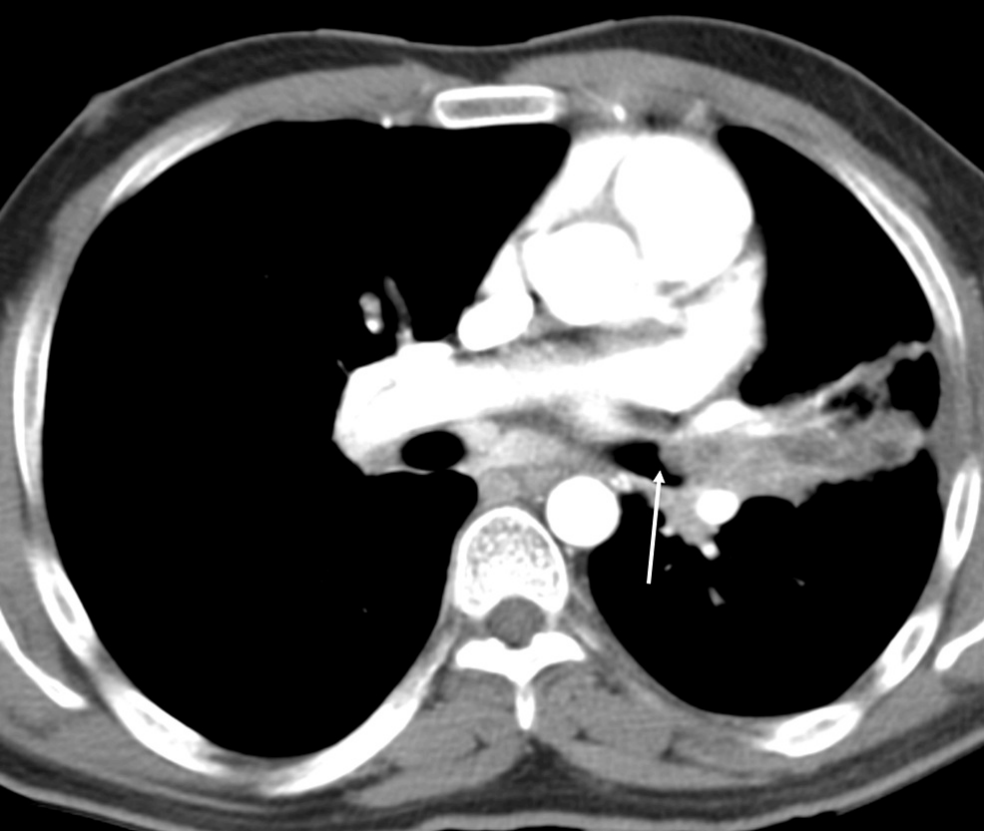
CECT showing volume loss in the LUL, endobronchial LUL lesion, mediastinal lymphadenopathy, and poorly defined low-density pulmonary nodules (October 21, 2011) CECT: contrast-enhanced computed tomography; LUL: left upper lobe

Transoral bronchoscopy revealed a polypoid, necrotic lesion within the left main stem bronchus. Biopsies were positive for a moderately differentiated, invasive squamous cell carcinoma (NSCLC). Due to the small sample size, molecular markers were not done. A CT thorax was performed, which confirmed volume loss in the LUL and characterized an endobronchial LUL lesion, mediastinal and hilar lymphadenopathy, and poorly defined low-density pulmonary nodules (Figure 2).

**Figure 3:**
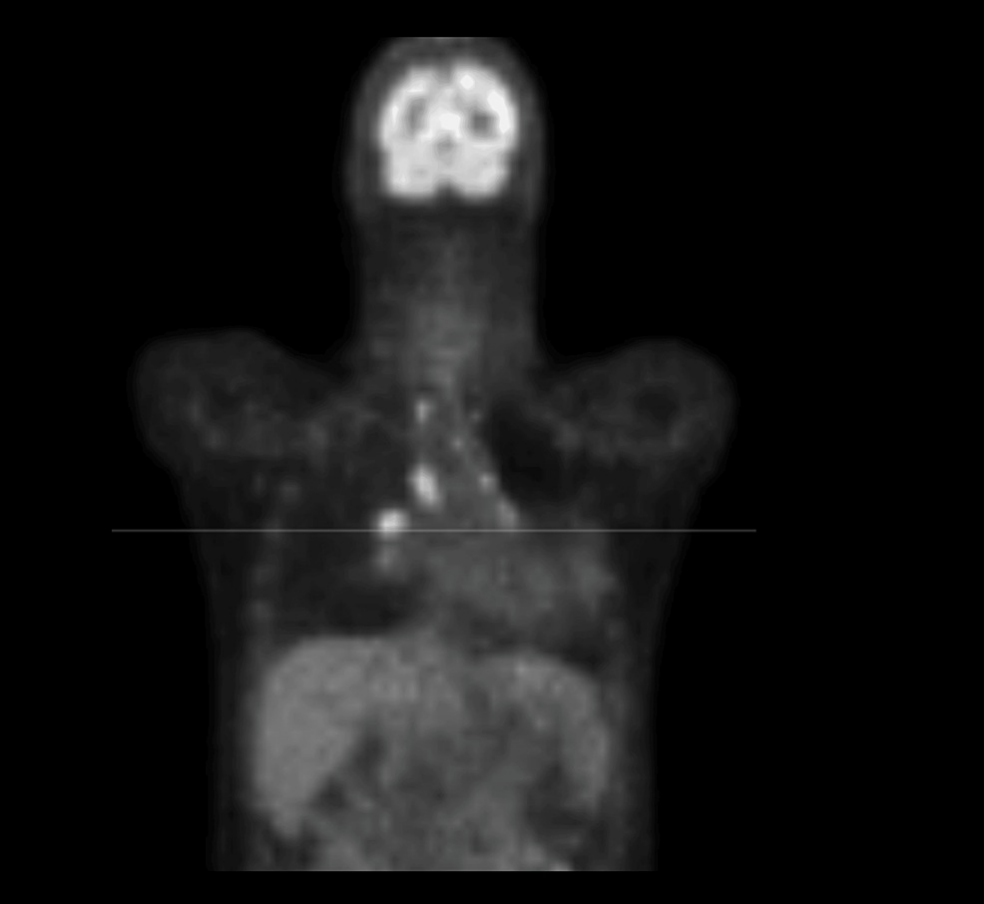
FDG-PET showing left bronchogenic carcinoma and contralateral hypermetabolic hilar adenopathy and upper mediastinal lymph nodes (October 26, 2011) FDG-PET: fluorodeoxyglucose-positron emission tomography

A positron emission tomography (PET) whole-body imaging was done in October 2011, which confirmed a left bronchogenic carcinoma and revealed contralateral hypermetabolic hilar adenopathy and contralateral hypermetabolic upper mediastinal lymph nodes (Figure 3).

The patient's TNM staging was T1N3M0. On PET, a focus of borderline hypermetabolism was identified in the right high cervical region just superior to the submandibular gland, which was suspected to reside within a level II lymph node. This last finding was thought to correspond to the patient’s lymphoma. In November 2011, a repeat endobronchial ultrasound (EBUS) was performed to identify if the mediastinal lymph nodes were lymphoma-related or lung cancer-related. However, the diagnosis was indeterminate and a lymphoproliferative process could not be excluded.

**Figure 4:**
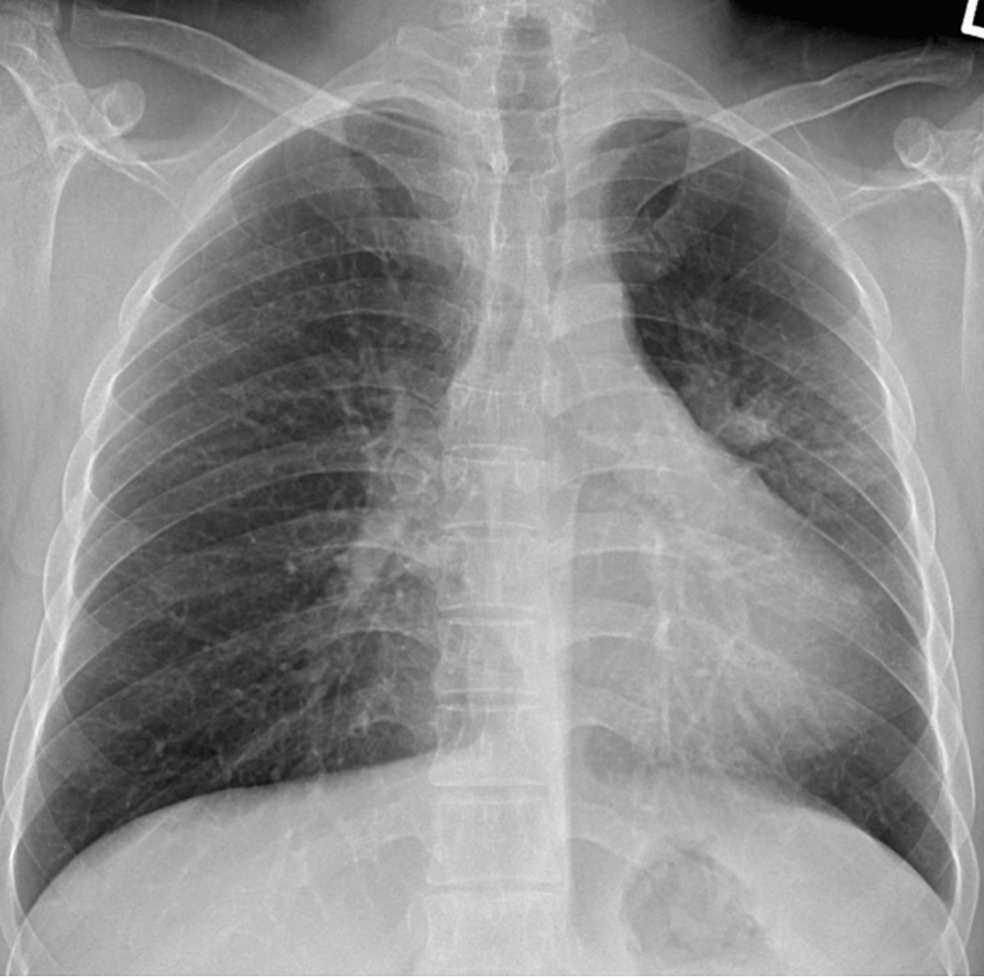
Chest X-ray of the LUL opacities showing no change (February 15, 2012) LUL: left upper lobe

In January 2012, the patient was seen at a thoracic surgery clinic where the surgeon determined that he was not suitable for surgical resection. The patient had significant underlying asthma (no evidence of COPD) and, due to his small stature (from BS), he also had reduced lung capacities. A multidisciplinary team meeting was conducted, and a decision to administer the patient a reduced dose of chemotherapy to accommodate for his BS was made, with the goal of shrinking the cancer area to prepare for radiation therapy. The treatment primarily focused on providing palliative care, as the patient was in bulky stage III of cancer. Between February 9 and March 30, 2012, the patient received weekly chemotherapy. He underwent 12 rounds of paclitaxel, with the dosage gradually increasing from 48 mg to 58 mg and finally to 72 mg. Around mid-February, a chest X-ray indicated some reduction in the consolidation in the left mid-zone, which was thought to be shrinkage of the radiation pneumonitis (Figure 4).

**Figure 5:**
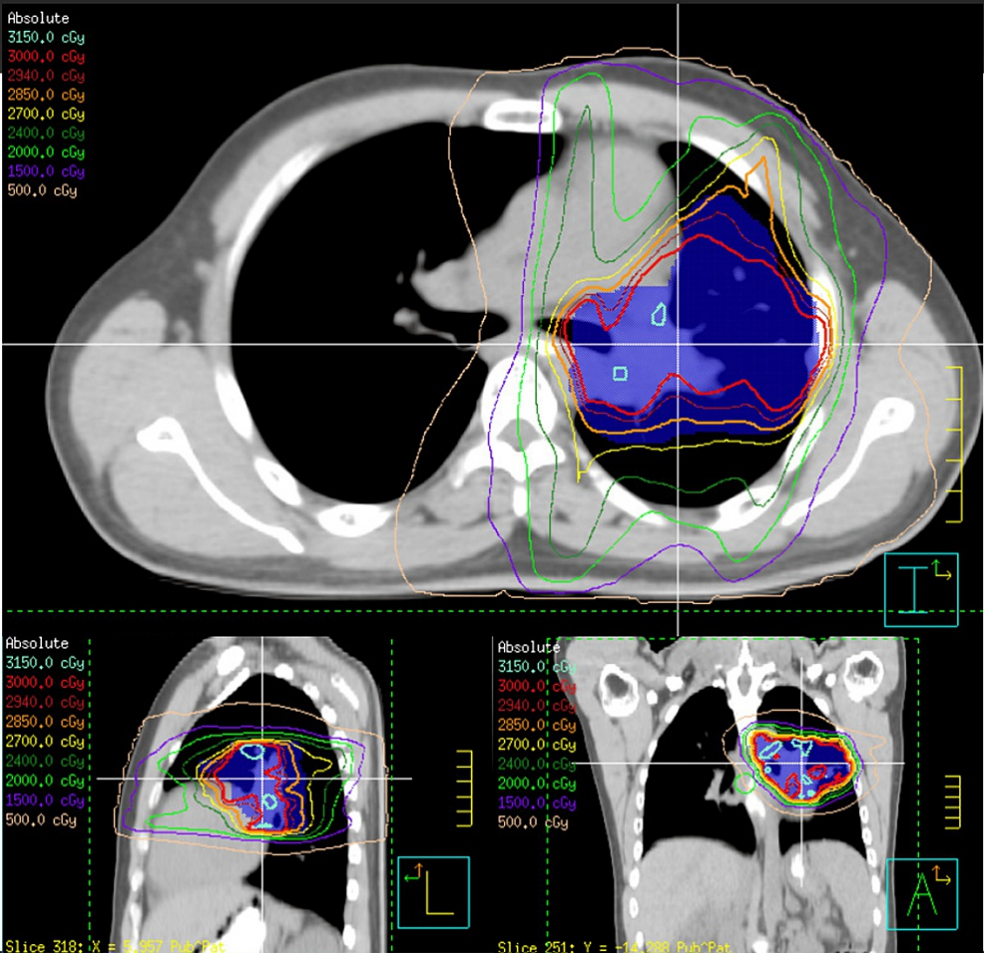
IMRT radiation plan IMRT: intensity-modulated radiation therapy

Subsequently, from April 10 to May 10, 2012, the patient commenced radiation therapy. He received 3000 cGY in 20 fractions. The radiation therapy was administered from five different angles using the intensity-modulated radiation therapy (IMRT) technique and was completed with good tolerance, providing good symptomatic relief for the patient (Table 1, Figure 5).

**Table 1:** Radiation dose volumes for different regions of interest (ROIs) GTV: gross tumor volume; ITV: internal target volume; PTV: planning target volume

Region of interest	Trial	Minimum dose (cGy)	Maximum dose (cGy)	Mean dose (cGy)	Standard deviation
Cord	3000_20	2.5	2427.7	358.3	686.7
GTV-0% phase	3000_20	2812.0	3247.3	3054.9	64.1
Heart	3000_20	30.4	3194.4	976.4	962.6
ITV nodal	3000_20	2881.9	3263.2	3109.0	69.9
ITV GTV	3000_20	2812.0	3247.3	3053.5	64.7
LUNG EVAL	3000_20	3.4	3158.5	553.4	882.5
Lung_Rt	3000_20	3.4	2319.0	141.3	177.7
PTV3000	3000_20	2377.1	3263.2	2999.0	106.4

**Figure 6:**
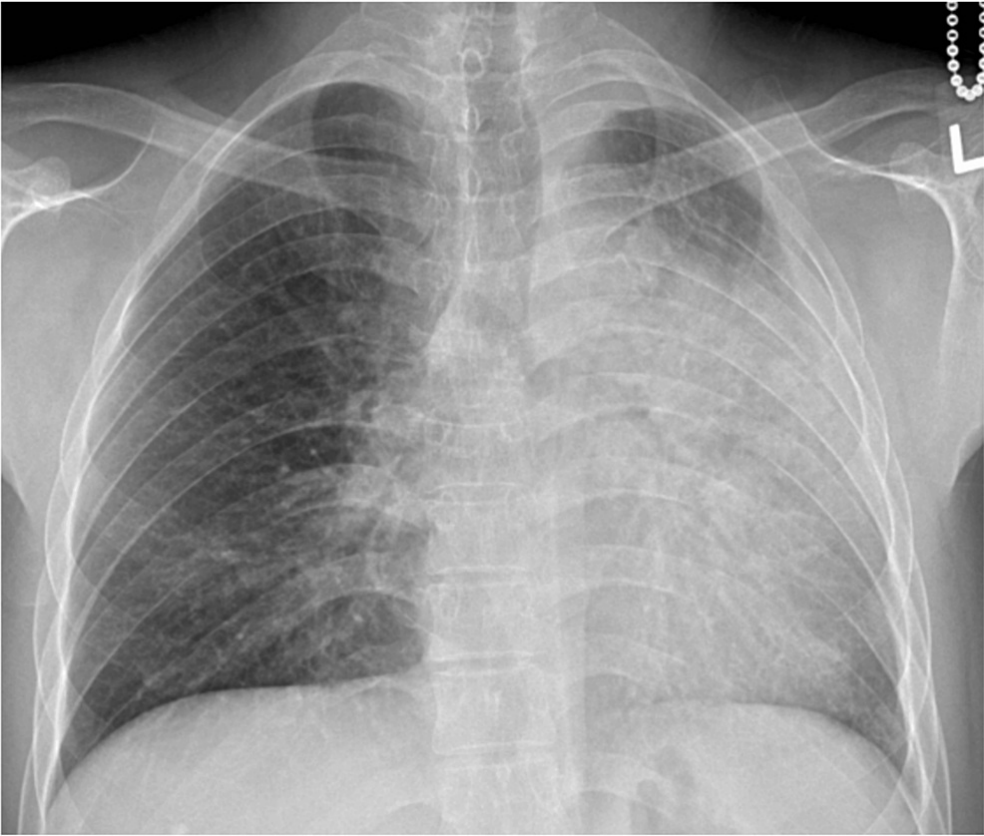
Chest X-ray showing LUL consolidation, possibly due to pneumonia or radiation (June 20, 2012; the last date of radiation was May 10) LUL: left upper lobe

By early June 2012, the X-rays did not show a significant change in the size of the tumor (Figure 6).

**Figure 7:**
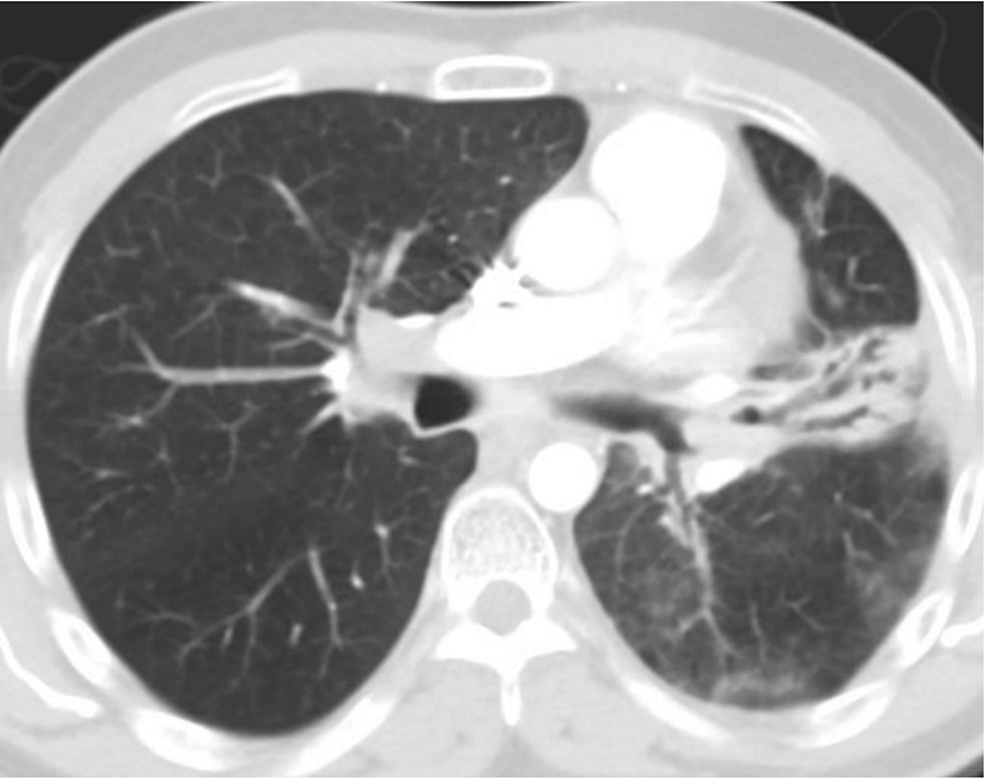
CT showing adenopathy at the hila has improved (June 12, 2012) CT: computed tomography

However, CT showed that adenopathy at the hila had improved. Specifically, the right hilar node had regressed from 3.0 x 1.6 cm down to 2.0 x 1.3 cm and the left lower paratracheal lymph node also improved, measuring approximately 11 x 16 mm, down from 17 x 22 mm previously (Figure 7).

While the palliative treatment course provided symptom relief to the patient, his cancer progressed over time, and he ultimately succumbed to it in April 2013 (18 months after the diagnosis of NSCLC). The cause of death was locally progressive lung cancer. No autopsy was performed.

## Discussion

Dr. David Bloom, a dermatologist, first described BS in 1954 [1]. Its clinical presentation includes short stature, learning disabilities, phototoxicity, and distinct facial features. Patients typically have a median lifespan of under 30 years and die primarily due to cancer. Managing the disease poses clinical challenges due to multiple comorbidities that worsen over time [8], such as COPD, diabetes, and immune deficiencies. It is a rare inherited genetic disorder with fewer than 300 cases currently reported worldwide [9]. The last Canadian case study on BS dates back to 1987, highlighting its rarity in Canada [10]. Our case presents a unique incidence of BS in a patient of Honduran ethnicity, a demographic from which there are no reported cases of the condition. The patient’s and his family’s lack of awareness of his illness, mental health issues, and other social factors further complicated the diagnosis of BS. With the occurrence of both B-cell lymphoma and NSCLC in our BS patient, a unique treatment regime was administered to prolong and enhance his quality of life.

The management of lymphoma in Bloom syndrome patients

Our case report details the management of a low-grade non-Hodgkin's B-cell lymphoma in a 34-year-old male with BS. Lymphoma is a common cancer that develops in patients with BS [6]. While there are no established guidelines on treating B-cell lymphoma in BS patients, several case reports have detailed the treatment of this condition in these patients with varying success [11,12,13]. The treatment regimens used include a rituximab-based chemotherapy protocol [11], high-dose methotrexate and high-dose cyclophosphamide [12], and R-CHOP (rituximab, cyclophosphamide, doxorubicin hydrochloride, vincristine sulfate, and prednisone) [13]. Further research is needed to delineate evidence-based guidelines for managing this common type of cancer in BS patients.

As detailed in our report, our patient’s molecular results, while not fully conclusive, were suggestive of a B-cell lymphoproliferative disorder. The early stage, and non-bulky presentation, compounded by the patient's significantly increased risk of lymphoma development due to his underlying BS led the hematology/oncology team to adopt a management strategy of surveillance alone. This treatment strategy was chosen to prevent unnecessary acute or late toxicity related to chemotherapy/radiotherapy to the patient. Also, given that individuals with BS have a heightened risk of second cancer development from chemotherapy/radiotherapy, this was an important consideration. The patient was regularly seen by the oncology team and surveillance testing included CT thorax/abdomen/pelvis taken periodically. The patient's lymphoma remained stable, never requiring active treatment. Thus, utilizing the treatment strategy of surveillance allowed us to spare the patient from unnecessary cytotoxicity for this condition. However, it should be noted that while our patient’s lymphoma did not progress, our management strategy may have enabled the progression of his disease.

The management of lung cancer in Bloom syndrome patients

In addition to lymphoma, our patient was also treated for an NSCLC that he subsequently developed. While cancer development in BS is common, lung cancer is one of the rarer types of cancer in this patient population, with only four cases recorded so far in the Malignant Neoplasms Diagnosed in Persons in the Bloom Syndrome Registry (1954-2022) [6]. As mentioned previously, the treatment for BS cancer patients is additionally complex as the current cancer needs to be treated without exposing patients unnecessarily to DNA-damaging agents that could precipitate future cancer development [6]. However, given that the goal for our patient was palliative treatment for his NSCLC, we were not as concerned about the long-term consequences of cytotoxicity.

Regarding chemotherapy, current weight-based regimens need to be adjusted in BS patients due to severe toxicity that can be life-threatening [14]. It is currently recommended that these cancer patients receive 50% or less of the standard chemotherapy dose. This dose adjustment has not been found to cause worse outcomes in BS patients but rather mitigates some of the toxicity risks [6]. In our patient, the chemotherapy agent paclitaxel was used as a single agent, which is not always tolerated well by BS patients. For example, a case report has described its poor tolerance in a 46-year-old BS woman with cervical cancer, causing 10% weight loss in three weeks, grade 1 asthenia, and persistent nausea [15]. The current dose of paclitaxel for NSCLC in a standard patient is 135 mg/m^2^ IV over 24 hours q3 weeks. We thus decided on a dosage regimen of 12 rounds of paclitaxel, with the dosage gradually increasing from 48 mg to 58 mg, and finally to 72 mg. This dose and regimen were well tolerated by our patient and his symptoms improved. However, there was no change in the tumor on imaging.

Standard radiation therapy is considered a high-risk treatment for patients with BS and thus should be avoided as far as possible [6]. Again, given that our patient’s treatment was palliative in intent, it was decided to use half the typical radiation dose to reduce the size of the NSCLC and improve his symptoms, delay the progression of his cancer, and improve his quality of life. Of note, a case report in the literature describes another patient with BS and NSCLC treated with standard dose radiation, which was well tolerated without additional cancer development or complications [16]. Our patient was treated with 3000 cGY in 20 fractions from five different angles using the IMRT technique (Figure 5). The dose and regimen were well tolerated by our patient and alleviated some of the bothersome symptoms of NSCLC, such as chest pain.

Overall, we recommend that clinicians consider using a modified treatment plan (possibly half the typical dose of chemotherapy and radiation therapy) when encountering a patient with a similar clinical presentation (BS diagnosis, stage III NSCLC at presentation, and palliative intent for treatment). In the future, molecular tumor markers may further enhance treatment outcomes in NSCLCs of various types [17].

## Conclusions

While cancer development in BS is very common due to the pathophysiology and radiosensitivity/chemosensitivity of the syndrome, it can be challenging to treat. Standard chemotherapy and radiotherapy doses are typically too toxic for this patient population as they create tolerance-related issues and can cause the future development of cancer and other complications. For the management of low-grade non-Hodgkin's B-cell lymphoma in a BS patient, the strategy of surveillance alone allowed us to spare the patient from unnecessary cytotoxicity as the cancer never progressed to a stage requiring active treatment. For the palliative treatment of stage III NSCLC in a BS patient, a chemotherapy regimen of paclitaxel and subsequently radiotherapy (3000 cGY in 20 fractions) was shown to aid in symptomatic palliation for our patient. Treatments were completed with good tolerance, with the main complaint being ongoing nausea and vomiting, which were managed through antiemetics and supportive care until the demise of the patient 18 months after the diagnosis of NSCLC. In the future, newer molecular agents may significantly contribute to cancer care in BS patients.
